# Patient preferences for whole-body MRI or conventional staging pathways in lung and colorectal cancer: a discrete choice experiment

**DOI:** 10.1007/s00330-019-06153-4

**Published:** 2019-04-01

**Authors:** Anne Miles, Stuart A. Taylor, Ruth E. C. Evans, Steve Halligan, Sandy Beare, John Bridgewater, Vicky Goh, Sam Janes, Neil Navani, Alf Oliver, Alison Morton, Andrea Rockall, Caroline S. Clarke, Stephen Morris, on behalf of the STREAMLINE investigators, A. Aboagye, L. Agoramoorthy, S. Ahmed, A. Amadi, G. Anand, G. Atkin, A. Austria, S. Ball, F. Bazari, R. Beable, H. Beedham, T. Beeston, N. Bharwani, G. Bhatnagar, A. Bhowmik, L. Blakeway, D. Blunt, P. Boavida, D. Boisfer, D. Breen, S. Burke, R. Butawan, Y. Campbell, E. Chang, D. Chao, S. Chukundah, B. Collins, C. Collins, V. Conteh, J. Couture, J. Crosbie, H. Curtis, A. Daniel, L. Davis, K. Desai, M. Duggan, S. Ellis, C. Elton, A. Engledow, C. Everitt, S. Ferdous, A. Frow, M. Furneaux, N. Gibbons, R. Glynne-Jones, A. Gogbashian, S. Gourtsoyianni, A. Green, Laura Green, Liz Green, A. Groves, A. Guthrie, E. Hadley, A. Hameeduddin, G. Hanid, S. Hans, B. Hans, A. Higginson, L. Honeyfield, H. Hughes, J. Hughes, L. Hurl, E. Isaac, M. Jackson, A. Jalloh, R. Jannapureddy, A. Jayme, A. Johnson, E. Johnson, P. Julka, J. Kalasthry, E. Karapanagiotou, S. Karp, C. Kay, J. Kellaway, S. Khan, D. Koh, T. Light, P. Limbu, S. Lock, I. Locke, T. Loke, A. Lowe, N. Lucas, S. Maheswaran, S. Mallett, E. Marwood, J. McGowan, F. Mckirdy, T. Mills-Baldock, T. Moon, V. Morgan, S. Nasseri, P. Nichols, C. Norman, E. Ntala, A. Nunes, A. Obichere, J. O’Donohue, I. Olaleye, A. Onajobi, T. O’Shaughnessy, A. Padhani, H. Pardoe, W. Partridge, U. Patel, K. Perry, W. Piga, D. Prezzi, K. Prior, S. Punwani, J. Pyers, H. Rafiee, F. Rahman, I. Rajanpandian, S. Ramesh, S. Raouf, K. Reczko, A. Reinhardt, D. Robinson, P. Russell, K. Sargus, E. Scurr, K. Shahabuddin, A. Sharp, B. Shepherd, K. Shiu, H. Sidhu, I. Simcock, C. Simeon, A. Smith, D. Smith, D. Snell, J. Spence, R. Srirajaskanthan, V. Stachini, S. Stegner, J. Stirling, N. Strickland, K. Tarver, J. Teague, M. Thaha, M. Train, S. Tulmuntaha, N. Tunariu, K. van Ree, A. Verjee, C. Wanstall, S. Weir, S. Wijeyekoon, J. Wilson, S. Wilson, T. Win, L. Woodrow, D. Yu

**Affiliations:** 1 Department of Psychological Sciences, Birkbeck, University of London, Malet Street, London, WC1E 7HX UK; 2 Centre for Medical Imaging, University College London, Charles Bell House, 43-45 Foley Street, London, W1W 7TS UK; 3 Cancer Research UK and University College London Clinical Trials Centre, 90 Tottenham Court Road, London, W1T 4TJ UK; 4 UCL Cancer Institute, Paul O Gorman Building, 72 Huntley Street London, London, WC1E 6DD UK; 5 Cancer Imaging, School of Biomedical Engineering and Imaging Sciences, King’s College London, Strand, London, WC2R 2LS UK; 6 Lungs for Living Research Centre, Division of Medicine, University College London, Gower Street, London, WC1E 6BT UK; 7 Department of Thoracic Medicine, UCLH and Lungs for Living Research Centre, University College London, London, WC1E 6BT UK; 8 National Cancer Research Institute, Angel Building, 407 St John Street, London, EC1V 4AD UK; 9 Department of Surgery and Cancer, Imperial College London, Kensington, London, SW7 2AZ UK; 10 Department of Radiology, Royal Marsden NHS Foundation Hospital Trust, Fulham Road, London, SW3 6JJ UK; 11 Research Department of Primary Care and Population Health, University College London, Upper Third Floor, UCL Medical School (Royal Free Campus), Rowland Hill Street, London, NW3 2PF UK; 12 Research Department of Applied Health Research, University College London, 1-19 Torrington Place, London, WC1E 6BT UK

**Keywords:** Magnetic resonance imaging, Cancer, Patient preference, Positron emission tomography, Tomography, X-ray computed

## Abstract

**Objectives:**

To determine the importance placed by patients on attributes associated with whole-body MRI (WB-MRI) and standard cancer staging pathways and ascertain drivers of preference.

**Methods:**

Patients recruited to two multi-centre diagnostic accuracy trials comparing WB-MRI with standard staging pathways in lung and colorectal cancer were invited to complete a discrete choice experiment (DCE), choosing between a series of alternate pathways in which 6 attributes (accuracy, time to diagnosis, scan duration, whole-body enclosure, radiation exposure, total scan number) were varied systematically. Data were analysed using a conditional logit regression model and marginal rates of substitution computed. The relative importance of each attribute and probabilities of choosing WB-MRI-based pathways were estimated.

**Results:**

A total of 138 patients (mean age 65, 61% male, lung *n* = 72, colorectal *n* = 66) participated (May 2015 to September 2016). Lung cancer patients valued time to diagnosis most highly, followed by accuracy, radiation exposure, number of scans, and time in the scanner. Colorectal cancer patients valued accuracy most highly, followed by time to diagnosis, radiation exposure, and number of scans. Patients were willing to wait 0.29 (lung) and 0.45 (colorectal) weeks for a 1% increase in pathway accuracy. Patients preferred WB-MRI-based pathways (probability 0.64 [lung], 0.66 [colorectal]) if they were equivalent in accuracy, total scan number, and time to diagnosis compared with a standard staging pathway.

**Conclusions:**

Staging pathways based on first-line WB-MRI are preferred by the majority of patients if they at least match standard pathways for diagnostic accuracy, time to diagnosis, and total scan number.

**Key Points:**

*• WB-MRI staging pathways are preferred to standard pathways by the majority of patients provided they at least match standard staging pathways for accuracy, total scan number, and time to diagnosis.*

*• For patients with lung cancer, time to diagnosis was the attribute valued most highly, followed by accuracy, radiation dose, number of additional scans, and time in a scanner. Preference for patients with colorectal cancer was similar.*

*• Most (63%) patients were willing to trade attributes, such as faster diagnosis, for improvements in pathway accuracy and reduced radiation exposure.*

**Electronic supplementary material:**

The online version of this article (10.1007/s00330-019-06153-4) contains supplementary material, which is available to authorized users.

## Introduction

Cancer staging pathways are complex, typically comprising a variety of imaging modalities including ultrasound, computed tomography (CT), and positron emission tomography (PET) CT. Multi-modality pathways are inconvenient for patients and prolong time to treatment. Conversely, whole-body magnetic resonance imaging (WB-MRI) may facilitate staging with a single investigation, while simultaneously achieving greater accuracy for metastatic disease, without imparting ionising radiation [1, 2]. However, patients perceive WB-MRI as more challenging than conventional staging scans [3], particularly among those with coexisting physical conditions and/or high anxiety levels [4]. MRI scan acquisition is noisy and whole-body imaging can take up to 1 h, much longer than standard CT or PET-CT. In addition, WB-MRI elicits claustrophobia in a substantial proportion of patients, which can terminate the scan prematurely [5]. Furthermore, WB-MRI may itself generate future tests such as PET-CT for equivocal findings.

Patients value staging accuracy highly [6] as well as rapid diagnosis [7]. The relative importance placed by patients on the comparative attributes of WB-MRI and standard staging pathways is unknown currently. For example, it is unclear what improvement in diagnostic accuracy patients would trade for lengthier scan times, or a longer wait before final diagnosis.

The aim of this study was to determine the relative importance placed by patients on a range of attributes associated with WB-MRI and standard staging pathways by performing a discrete choice experiment and to ascertain which of these attributes govern patient preferences for one pathway over the other.

## Materials and methods

Discrete choice experiments (DCE) elicit preferences by asking individuals to indicate their choice between two or more options, where each option contains characteristics or attributes (e.g. scan accuracy, scan duration) that are varied and are differentiated by values or levels of each attribute. By analysing the choices people make, the relative importance of different attributes can be determined. The international DCE guidelines were followed for study design and analysis [8–10].

### Patients and recruitment

Recruitment took place within the context of two prospective, multi-centred cohort trials investigating the diagnostic accuracy and cost-effectiveness of WB-MRI compared with standard pathways for staging newly diagnosed lung and colorectal cancers (‘Streamline L’ and ‘Streamline C’). The trial protocols have been published previously [11]. For Streamline L, patients were recruited from 16 hospitals and underwent WB-MRI at one of seven centres. For Streamline C, patients were recruited from 16 hospitals and underwent WB-MRI at one of eight centres. Across both trials, WB-MRI was performed on scanners from three major vendors.

Recruits underwent WB-MRI (the research intervention) in addition to conventional staging scans. The WB-MRI scans were performed according to a minimum dataset, including axial whole-body (vertex to mid-thigh) axial diffusion and axial T2- and T1 (pre- and post-intravenous gadolinium-containing contrast medium)-weighted imaging. A Dixon sequence was used if available on the scanner. Slice thickness was between 5 and 7 mm and post gadolinium images were acquired at a minimum through the liver (portal phase), lung (equilibrium phase), and brain. Exact parameters differed between sites, but all sites utilised protocols that could be completed in 1 h or less.

**Fig. 1:**
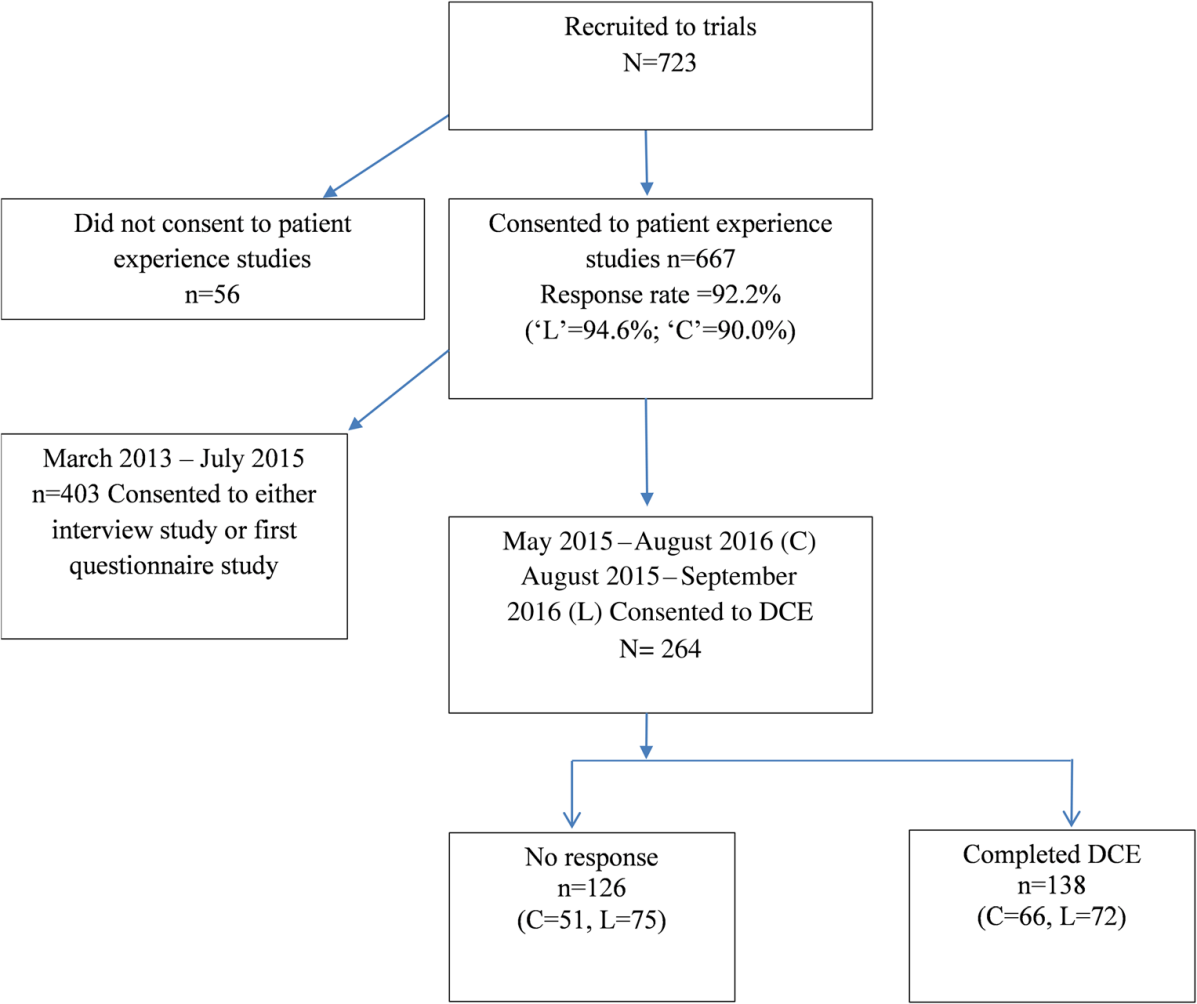
Flow diagram of participants through the study (May 2015–September 2016)

Patients recruited to the Streamline trials were initially invited to either an interview [3] or questionnaire study (both aimed at assessing patients’ experience of staging scans) [4] (Fig. 1). Once recruitment to these studies was complete, patients were exclusively invited to complete the current DCE study [11].

Ethical approval for this study was granted by the Camden and Islington NRES committee on 03/10/2012, project numbers: 12/LO/1176 (Streamline C) and 12/LO/1177 (Streamline L). Participants gave written informed consent for participation in the DCE study.

DCE questionnaires were posted to patients by the Clinical Trials Unit within 1–2 days of consenting to trial participation and while they were still undergoing staging. Patients were provided with stamped addressed reply envelopes and were paid £20 upon receipt of a completed questionnaire. Consecutive patients were approached to participate until a minimum of 42 patients had returned a questionnaire for each cancer type cohort (see power calculation supplementary data).

### Attributes and levels

DCE attributes were selected by study investigators to capture known or potential important differences between WB-MRI and standard staging pathways; these were informed by findings from the patient interview and questionnaire studies described above [3, 4]. The Streamline trials were designed to determine whether WB-MRI is more accurate than standard staging pathways for detecting metastatic disease, while simultaneously decreasing the number of individual scans, thereby reducing time to diagnosis. Accordingly, accuracy, scan number, and time to diagnosis were selected as potentially important attributes. In addition, the following attributes were also included, having also been identified as potentially important: scan duration, need for the whole body and head to be enclosed by the scanner, and increased cancer risk due to exposure to ionising radiation.

Credible levels for each attribute were chosen based on either known characteristics, such as scan duration, or after appropriate literature review, for example radiation exposure and scan/pathway accuracy [12–14]. The number of scans in each pathway required to reaching a final diagnosis was based on typical staging pathways, supported by data accrued during the main trials.

Attributes and levels are summarised in Table 1.

**Table 1:** Attributes and attribute levels

Attributes	Attribute levels		
Time in a scanner	10 min	30 min	60 min
Time to reach a final diagnosis	1 week	3 weeks	5 weeks
Associated increase in cancer risk due to radiation exposure	None	1 in 1000 risk of cancer	2 in 1000 risk of cancer
Number of additional staging scans before final diagnosis	0	1	2
Accuracy for metastatic disease (%)	85	90	95
Need for whole body and head to be in a scanner	No	Yes	–

### Questionnaire design

Of the six attributes, five had three levels and one had two levels. The total number of attribute combinations was therefore 486 (= 3^5^ × 2^1^). Each question presented patients with a binary choice set (pathway A vs. pathway B), resulting in a possible 235,710 choices (= 486 × 485). To reduce the number of choices to a manageable number, an orthogonal fractional main effects design was applied for pathway A [15]. Pathway B was generated by shifting the attribute level up by one category for each attribute (e.g. if the time in a scanner was 10 [30] {60} min in Pathway A then it was shifted to 30 [60] {10} min in Pathway B). We reduced the number of choice sets to 18, which were split into two blocks of nine, and half the respondents in each group were assigned to each block. Patients were randomly assigned to complete either choice sets 1 to 9 (Questionnaire A) or 10 to 18 (Questionnaire B) and asked to complete all 9 choice sets. A similar approach has been used in previous DCE studies, balancing the desire to include more choice sets to cover a wider number of attribute combinations against respondent burden [16]. The choice sets were presented in a random order within each questionnaire. We did not include an opt-out or ‘neither’ option as patients recruited to the Streamline trials were unlikely to choose not to undergo staging. Prior to administering the DCE questionnaire, its burden and content were reviewed and modified for clarity by the Streamline trial management group, which included 2 patient representatives.

**Fig. 2:**
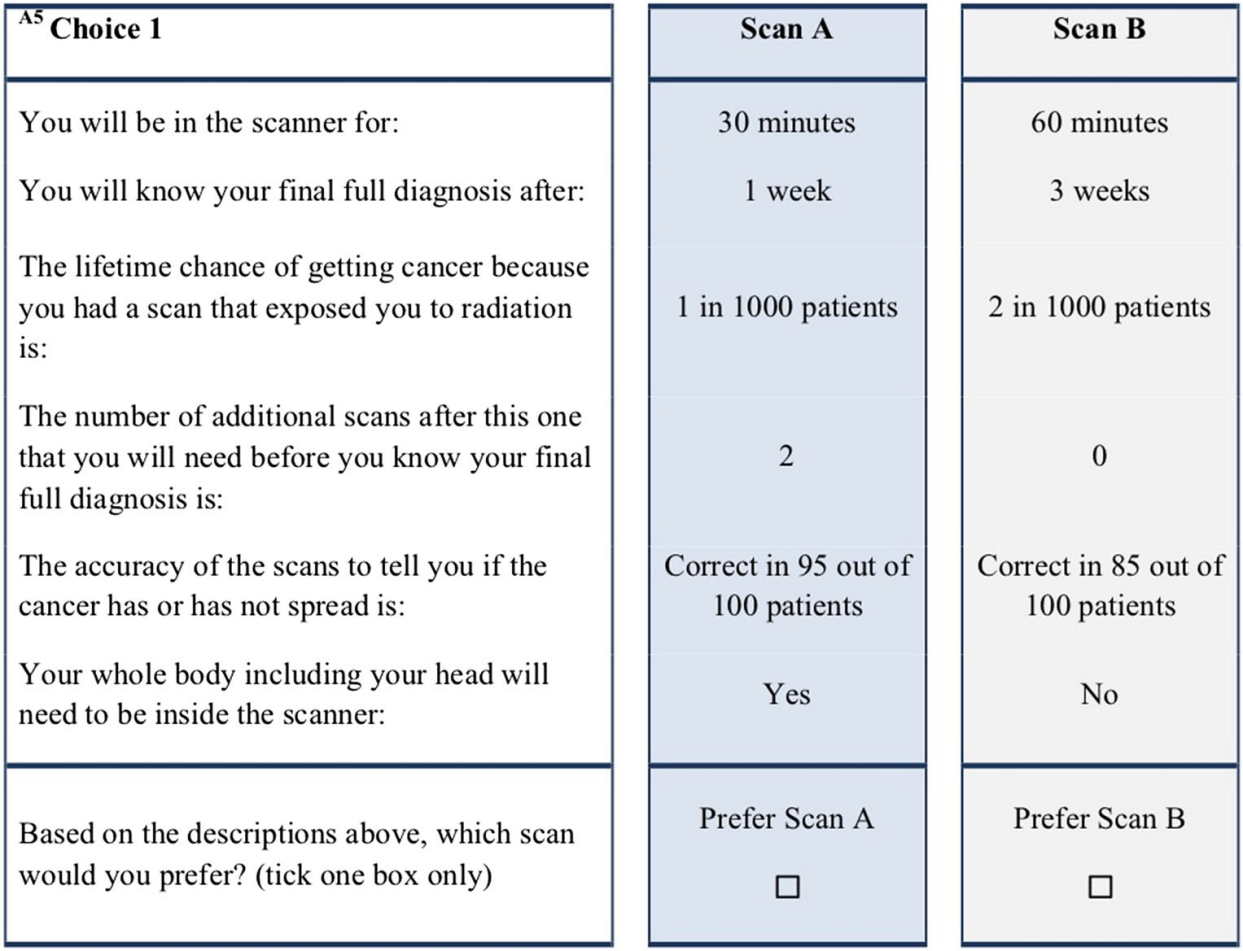
Example of a choice set

An example of a choice set is shown in Fig. 2.

A range of demographic and health-related variables were also collected from participants (see Questionnaire supplementary data), along with self-rated health, presence of comorbidities, and positive and negative mood (using the PANAS, phrased to ask about current mood [17, 18] and whether patients had already had a WB-MRI at the time of completing the questionnaire). Missing data for age and gender were populated with data from the main trial (with patient consent).

Participants were also asked whether they preferred WB-MRI or standard tests (“If you had to have JUST ONE of the tests which one would you prefer?”).

An example administered questionnaire (Questionnaire A for lung cancer patients) is shown in supplementary data.

### Analysis

The analysis is described in detail in supplementary data.

In brief, DCE data were analysed using a conditional logit regression model (fixed effects logit) where the outcome was the test preference (scan A or B) and the variables in the equation were the individual attributes. We undertook exploratory analyses to investigate whether within each cohort preferences varied by sample sub-groups. We conducted likelihood ratio tests to test the null hypothesis that none of the attributes were related to preferences.

The relative importance of each attribute was calculated as the difference in preference weights between the best or most preferred level of each attribute and the worst or least preferred level of the same attribute [19].

We used the regression coefficients to compute marginal rates of substitution (MRS). The MRS allows direct assessment of how much of one attribute participants are willing to trade for one unit of another attribute and therefore enables a comparison of different attributes on a common scale.

**Fig. 3:**
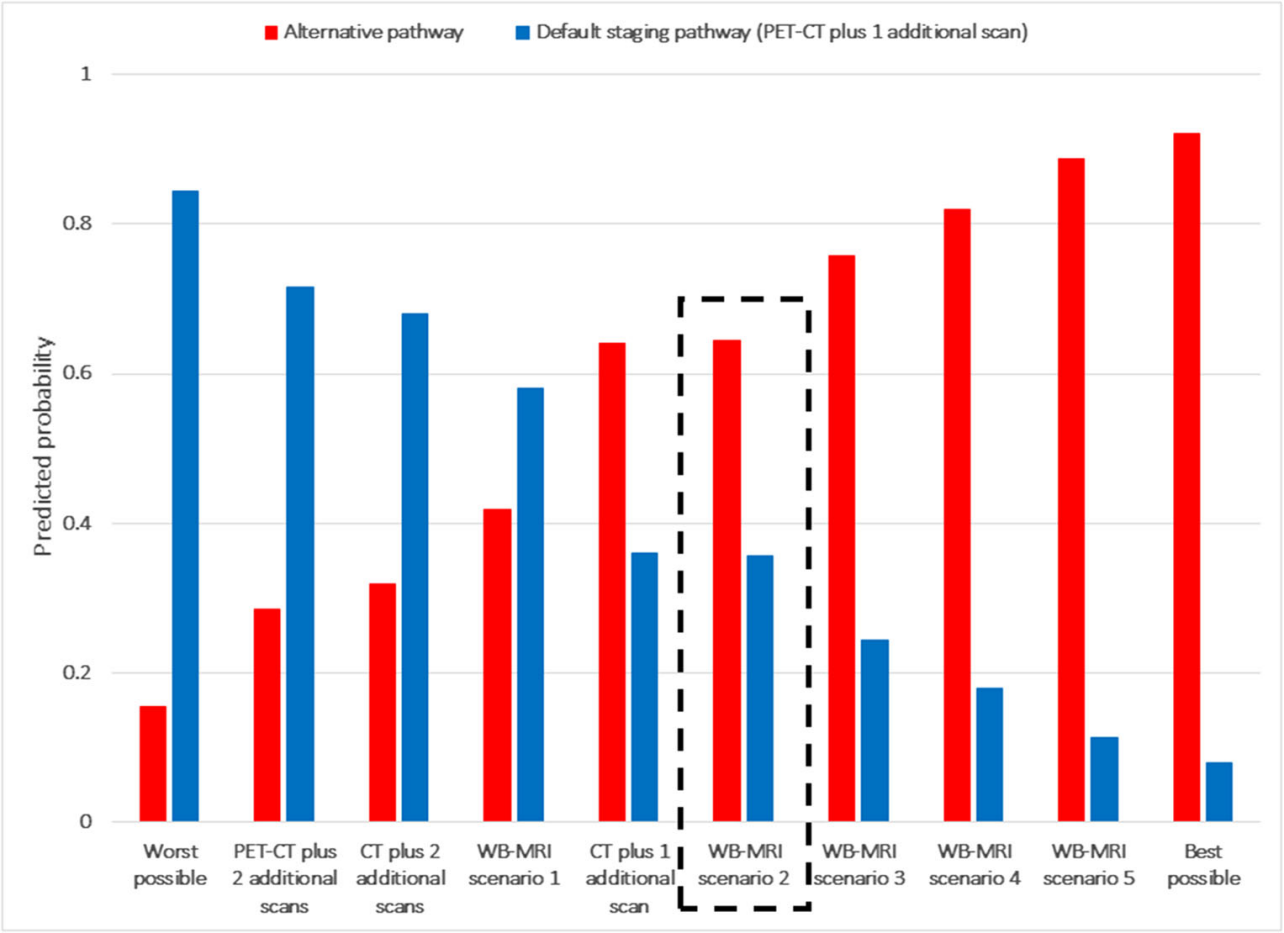
Predicted probabilities of choosing an alternate staging pathways in comparison to a default staging pathway (PET-CT plus one additional scan) (lung cancer patients). *Description of tests*: Default staging pathway (PET-CT plus 1 additional scan) in every case: 30-min time in a scanner, 3 weeks to diagnosis, 2/1000 cancer risk due to radiation dose, 1 additional scan, 90% accuracy, no need for whole body and head to be in a scanner. Worst possible test: 60-min time in a scanner, 5 weeks to diagnosis, 2/1000 cancer risk due to radiation dose, 2 additional scans, 85% accuracy, need for whole body and head to be in a scanner. PET-CT plus 2 additional scans: 30-min time in a scanner, 5 weeks to diagnosis, 2/1000 cancer risk due to radiation dose, 2 additional scans, 90% accuracy, no need for whole body and head to be in a scanner. CT plus 2 additional scans: 10-min time in a scanner, 5 weeks to diagnosis, 2/1000 cancer risk due to radiation dose, 2 additional scans, 90% accuracy, no need for whole body and head to be in a scanner. WB-MRI scenario 1: longer scan time, no radiation, whole body enclosed, longer time to diagnosis, more scans = 60-min time in a scanner, 5 weeks to diagnosis, 0/1000 cancer risk due to radiation dose, 2 additional scans, 90% accuracy, need for whole body and head to be in a scanner. CT plus 1 additional scan: 10 min time in a scanner, 3 weeks to diagnosis, 1/1000 cancer risk due to radiation dose, 1 additional scan, 90% accuracy, no need for whole body and head to be in a scanner. WB-MRI scenario 2: longer scan time, no radiation, whole body enclosed = 60-min time in a scanner, 3 weeks to diagnosis, 0/1000 cancer risk due to radiation dose, 1 additional scan, 90% accuracy, need for whole body and head to be in a scanner. WB-MRI scenario 3: longer scan time, no radiation, whole body enclosed, more accurate = 60-min time in a scanner, 3 weeks to diagnosis, 0/1000 cancer risk due to radiation dose, 1 additional scan, 95% accuracy, need for whole body and head to be in a scanner. WB-MRI scenario 4: longer scan time, no radiation, whole body enclosed, quicker time to diagnosis, fewer scans = 60-min time in a scanner, 1 week to diagnosis, 0/1000 cancer risk due to radiation dose, 0 additional scans, 90% accuracy, need for whole body and head to be in a scanner. WB-MRI scenario 5: longer scan time, no radiation, whole body enclosed, more accurate, quicker time to diagnosis, fewer scans = 60-min time in a scanner, 1 week to diagnosis, 0/1000 cancer risk due to radiation dose, 0 additional scans, 95% accuracy, need for whole body and head to be in a scanner. Best possible pathway: 10-min time in a scanner, 1 week to diagnosis, 0/1000 cancer risk due to radiation dose, 0 additional scans, 95% accuracy, no need for whole body and head to be in a scanner. The comparison indicated by the dashed box (WB-MRI scenario 2) is one in which WB-MRI differs from the default staging pathway according to established differences (time in a scanner, exposure to ionising radiation, need for the whole body and head to be inside the scanner) but for which other attributes (time to diagnosis, number of additional scans, accuracy) are assumed to be the same between the two pathways

**Fig. 4:**
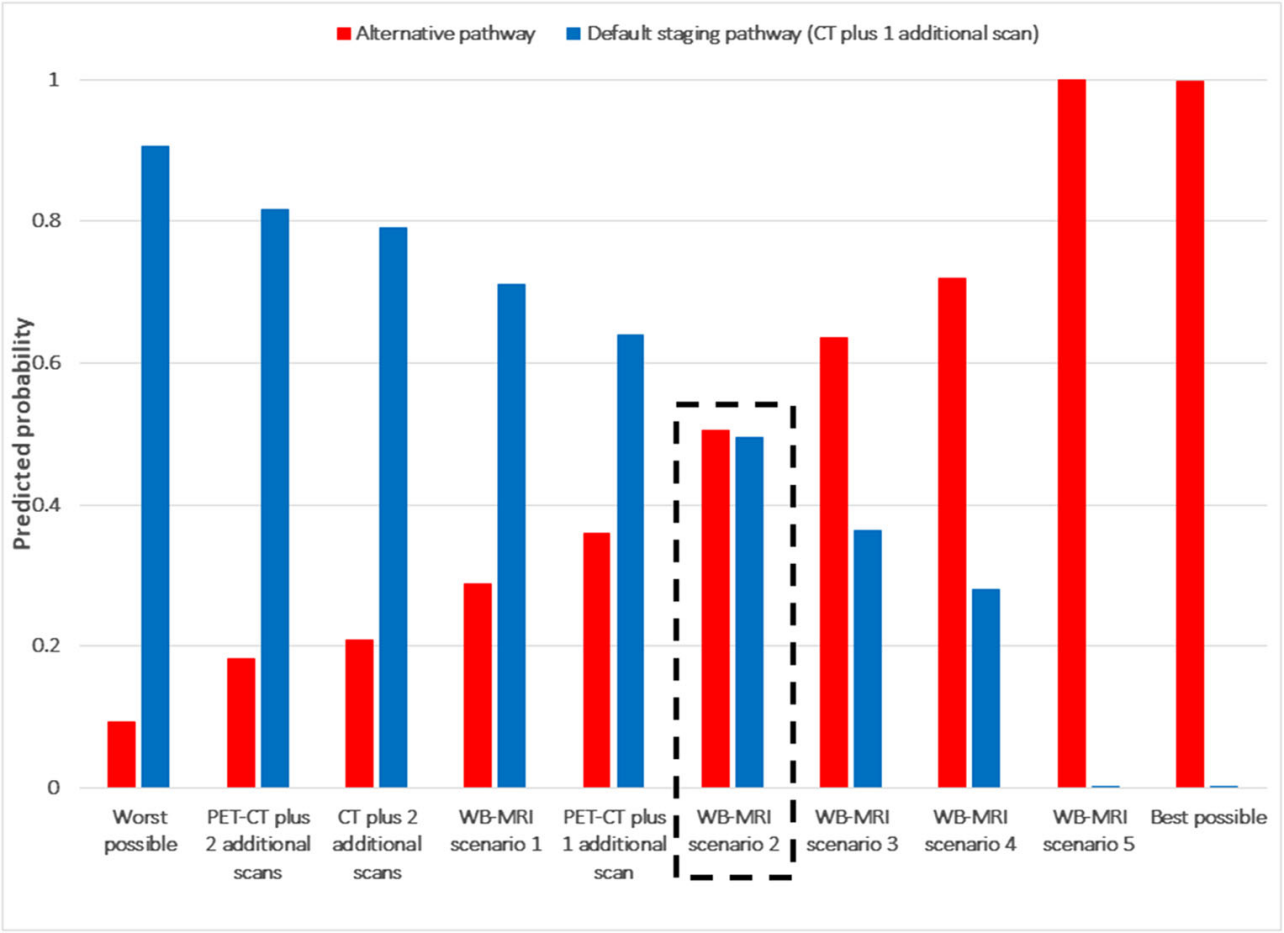
Predicted probabilities of choosing an alternate staging pathways in comparison to a default staging pathway (CT plus one additional scan) (colorectal cancer patients). *Description of tests*: Default staging pathway (CT plus 1 additional scan) in every case: 10-min time in a scanner, 3 weeks to diagnosis, 1/1000 cancer risk due to radiation dose, 1 additional scan, 90% accuracy, no need for whole body and head to be in a scanner. Worst possible pathway: 60-min time in a scanner, 5 weeks to diagnosis, 2/1000 cancer risk due to radiation dose, 2 additional scans, 85% accuracy, need for whole body and head to be in a scanner. PET-CT plus 2 additional scans: 30-min time in a scanner, 5 weeks to diagnosis, 2/1000 cancer risk due to radiation dose, 2 additional scans, 90% accuracy, no need for whole body and head to be in a scanner. CT plus 2 additional scans: 10-min time in a scanner, 5 weeks to diagnosis, 2/1000 cancer risk due to radiation dose, 2 additional scans, 90% accuracy, no need for whole body and head to be in a scanner. WB-MRI scenario 1: longer scan time, no radiation, whole body enclosed, longer time to diagnosis, more scans = 60-min time in a scanner, 5 weeks to diagnosis, 0/1000 cancer risk due to radiation dose, 2 additional scans, 90% accuracy, need for whole body and head to be in a scanner. PET-CT plus 1 additional scan: 30-min time in a scanner, 3 weeks to diagnosis, 2/1000 cancer risk due to radiation dose, 1 additional scan, 90% accuracy, no need for whole body and head to be in a scanner. WB-MRI scenario 2: longer scan time, no radiation, whole body enclosed = 60-min time in a scanner, 3 weeks to diagnosis, 0/1000 cancer risk due to radiation dose, 1 additional scan, 90% accuracy, need for whole body and head to be in a scanner. WB-MRI scenario 3: longer scan time, no radiation, whole body enclosed, more accurate = 60-min time in a scanner, 3 weeks to diagnosis, 0/1000 cancer risk due to radiation dose, 1 additional scan, 95% accuracy, need for whole body and head to be in a scanner. WB-MRI scenario 4: longer scan time, no radiation, whole body enclosed, quicker time to diagnosis, fewer scans = 60-min time in a scanner, 1 week to diagnosis, 0/1000 cancer risk due to radiation dose, 0 additional scans, 90% accuracy, need for whole body and head to be in a scanner. WB-MRI scenario 5: longer scan time, no radiation, whole body enclosed, more accurate, quicker time to diagnosis, fewer scans = 60-min time in a scanner, 1 week to diagnosis, 0/1000 cancer risk due to radiation dose, 0 additional scans, 95% accuracy, need for whole body and head to be in a scanner. Best possible pathway: 10-min time in a scanner, 1 week to diagnosis, 0/1000 cancer risk due to radiation dose, 0 additional scans, 95% accuracy, no need for whole body and head to be in a scanner. The comparison indicated by the dashed box (WB-MRI scenario 2) is one in which WB-MRI differs from the default staging pathway according to established differences (time in a scanner, exposure to ionising radiation, need for the whole body and head to be inside the scanner) but for which other attributes (time to diagnosis, number of additional scans, accuracy) are assumed to be the same between the two pathways

We also used the regression analysis results to calculate the predicted probabilities of choosing alternative pathways (for example based on WB-MRI), compared with a default standard staging pathway The selected default standard pathway was PET-CT plus one additional scan (lung cancer), or CT plus 1 additional scan (colorectal cancer) (Figs. 3 and 4).

We compared default staging pathways to alternative pathways with varying attribute levels based around PET-CT, CT, and WB-MRI. We considered several scenarios for WB-MRI-based pathways, although fixed the following attributes: (i) 60 min in the scanner, (ii) no risk of cancer from radiation exposure, and (iii) requirement for the whole body and head to be enclosed. We then varied combinations of time to diagnosis, number of additional scans, and accuracy of WB-MRI individually and jointly. Non-traders were included in the analysis.

All data were analysed using SPSS version 24 and Stata version 13.

## Results

### Participants

One hundred thirty-eight patients completed part or all of the questionnaires, 72 recruited to Streamline L, and 66 recruited to Streamline C. A total of 128 completed all 9 choice sets (66 in Streamline L, 62 in Streamline C). Demographic data are shown in Table 2. Most patients had already undergone WB-MRI at the time of completing the DCE (113 [86%] of 131 answering the question), with no significant difference between the cohorts (Streamline C, 55/64, 86%; Streamline L, 58/67, 87%).

**Table 2:** Demographic and psychological characteristics of the sample. Numbers are *N* (percent) unless stated otherwise

	All patients	Lung cancer patients	Colorectal cancer patients	Group differences^1^
Demographics				
Age^a^ (mean (SD))	64.7 (10.9) (*n* = 138)	66.0 (10.8) (*n* = 72)	63.2 (10.9) (*n* = 66)	*p* = 0.122
Male gender^a^	84 (60.9) (*n* = 138)	42 (59.2) (*n* = 72)	41 (62.1) (*n* = 66)	*p* = 0.723
White ethnicity^b^	112 (84.2) (*n* = 133)	59 (85.5) (*n* = 69)	53 (82.8) (*n* = 64)	*p* = 0.670
Educational qualifications^c^				
None	35 (28.2)	28 (44.4)	7 (11.5)	*p* < 0.001
Below degree level	39 (31.5)	17 (27.0)	22 (36.1)	
Degree level or equivalent	50 (40.3) (*n* = 124)	18 (28.6) (*n* = 63)	32 (52.5) (*n* = 61)	
Home ownership (yes)^c^	82 (64.6) (*n* = 127)	34 (50.0) (*n* = 68)	48 (81.4) (*n* = 59)	*p* < 0.001
Car ownership (yes)^c^	114 (87.0) (*n* = 131)	61 (88.4) (*n* = 69)	53 (85.5) (*n* = 62)	*p* = 0.619
Marital status^b^				
Married/cohabiting	85 (63.4)	42 (58.3)	43 (69.4)	*p* = 0.403
Single	22 (16.4)	13 (18.1)	9 (14.5)	
Divorced, separated, widowed	27 (20.1) (*n* = 134)	17 (23.6) (*n* = 72)	10 (16.1) (*n* = 62)	
Employment status^b^				
Employed full-time, part-time, self-employed, full-time homemaker	46 (34.1)	17 (23.9)	29 (45.3)	*p* = 0.018
Retired	74 (54.8)	43 (60.6)	31 (48.4)	
Unemployed, disabled, or too ill to work	15 (11.1) (*n* = 135)	11 (15.5) (*n* = 71)	4 (6.3) (*n* = 64)	
Health				
Self-rated health^b^				
Very bad, bad, or fair	60 (44.1)	41 (57.7)	19 (29.2)	*p* = 0.001
Good or very good	76 (55.9) (*n* = 136)	30 (42.3) (*n* = 71)	46 (70.8) (*n* = 65)	
Presence of comorbidities^a^	77 (55.8) (*n* = 138)	48 (66.7) (*n* = 72)	29 (43.9) (*n* = 66)	*p* = 0.007
Psychological variables				
Negative mood^b^ (mean (SD))	18.01 (7.45) (*n* = 136)	18.76 (7.62) (*n* = 71)	17.20 (7.23) (*n* = 65)	*p* = 0.224
Positive mood^b^ (mean (SD))	27.32 (7.92) (*n* = 137)	25.73 (7.72) (*n* = 71)	29.03 (7.84) (*n* = 66)	*p* = 0.014

^a^No missing data

^b^Missing data less than 5%

^c^Missing data greater than 5%

^1^Patients recruited to Streamline C were more likely to have educational qualifications, own a home, and be in employment than patients recruited to Streamline L. They were less likely to report comorbidities and more likely to rate their current health as good or very good and report higher levels of positive mood than Streamline L patients

### Regression analysis

Likelihood ratio tests rejected the null hypothesis that none of the attributes were related to preferences (Table 3). Overall, participants preferred (i) to wait less time for a diagnosis, (ii) a lower dose of radiation exposure, (iii) fewer additional scans, and (iv) greater test accuracy. Conditional on these factors, preferences were not influenced significantly by time in the scanner or the need for the whole body and head to be enclosed. Preferences differed significantly between lung cancer and colorectal cancer patients. Time in the scanner did significantly influence the preferences of lung cancer patients. Both cohorts preferred tests with higher accuracy, but the preference was significantly greater for patients with colorectal cancer (*p* = 0.03). For the other attributes, preferences were not significantly different between the two cohorts.

**Table 3:** Results of conditional logit regression analysis by group

		All patients		Lung cancer patients		Colorectal cancer patients		
Attributes	Levels	Coefficient (95% CI)	RI	Coefficient (95% CI)	RI	Coefficient (95% CI)	RI	*p* value^b^
Time in a scanner	Minutes	− 0.002 (− 0.007, 0.002)^a^	–	− 0.008 (− 0.014, − 0.002)	0.04	0.005 (− 0.002, 0.012)^a^	–	0.01
Time to diagnosis	Weeks	− 0.355 (− 0.411, − 0.300)	1.42	− 0.372 (− 0.449, − 0.295)	1.49	− 0.349 (− 0.432, − 0.265)	1.40	0.70
Radiation dose	Risk of cancer (/1000)	− 0.421 (− 0.521, − 0.320)	0.84	− 0.413 (− 0.551, − 0.274)	0.83	− 0.436 (− 0.587, − 0.286)	0.87	0.83
Number of additional scans	Number	− 0.192 (− 0.299, − 0.084)	0.38	− 0.179 (− 0.330, − 0.028)	0.36	− 0.224 (− 0.382, − 0.067)	0.45	0.69
Accuracy	Percentage	0.128 (0.107, 0.150)	1.28	0.109 (0.079, 0.138)	1.09	0.156 (0.122, 0.190)	1.56	0.03
Need for whole body and head to be in a scanner	No	–	–	–	–	–	–	
	Yes	0.020 (− 0.129, 0.170)^a^		0.017 (−0.190, 0.224)^a^		− 0.007 (− 0.233, 0.220)^a^		0.88
Observations/respondents		2362/138		1230/72		1132/66		0.02
Likelihood ratio *χ*^2^ (*p* value)		445.5 (< 0.01)		221.9 (< 0.01)		238.9 (< 0.01)		

NB: Different attributes do not have the same unit of change so cannot be directly compared with one another

*CI*, confidence interval; *RI*, relative importance (see text); RI is calculated for attributes with coefficients that were significantly different from zero

^a^Coefficient not significantly different from zero; all other coefficients significant at *p* value < 0.05

^b^*p* values are from *χ*^2^ tests that coefficients are equal for lung cancer and colorectal cancer patients. *p* values < 0.05 indicate coefficients are significantly different between groups. *p* value in the bottom row is for joint test across all coefficients

### Relative importance of the attributes

Over the range of levels included in the study, for patients with lung cancer, time to diagnosis was the attribute valued most highly, followed by accuracy, radiation dose, number of additional scans, and time in a scanner (Table 3). For patients with colorectal cancer, accuracy was valued most highly, followed by time to diagnosis, radiation dose, and number of additional scans.

In exploratory analyses, within each cohort, there were no significant differences in preferences according to sub-groups stratified by gender, age, comorbidities, employment status, marital status, and positive mood. For patients with lung cancer (but not colorectal), there were significant variations when patients were stratified by home ownership, education, and self-rated health (supplementary data, Tables A1 to A3). For example, the influence of diagnostic accuracy on preferences was greater for lung cancer patients who were home-owning or had higher self-rated health.

Overall, 32/59 (54.2%) lung cancer patients and 45/61 (73.8%) colon cancer patients who answered the question selected WB-MRI over standard scans. There were no significant differences in attribute preferences between colorectal cancer patients who preferred WB-MRI compared with those who stated a preference for standard staging scans. Conversely, in patients with lung cancer, those stating an overall preference for standard staging scans preferred less time in a scanner and to not have their whole body and head enclosed (supplementary data, Table A4).

### Traders vs non-traders

Thirty-seven percent (*n* = 51/138) of patients were ‘non-traders’ (non-traders are participants whose preferences are determined by a single attribute, which they do not trade-off against any of the other attributes presented; suppose for example that a respondent was a non-trader with respect to the ‘time to reach a final diagnosis’ attribute, this would mean they would always select the pathway with the lowest time to reach a final diagnosis, irrespective of the levels of any of the other attributes). The most common attributes patients would not trade were higher accuracy, faster time to diagnosis, and reduced cancer risk due to scan-related radiation exposure (see supplementary data, Table A5).

### Marginal rates of substitution

Table 4 shows results of the MRS analysis. Lung cancer patients were willing to wait just over 1 extra week (MRS = − 1.11) in return for a 1 in 1000 reduction in the risk of cancer from radiation exposure. They were willing to wait around an extra half a week (MRS = − 0.48) to avoid an additional scan and around a third of a week (MRS = 0.29) for every 1% increase in accuracy (i.e. 1.45 weeks for a 5% increase in accuracy). The willingness to wait longer for a diagnosis for a reduction in the time in a scanner was negligible (− 0.02). These figures were broadly similar to colorectal cancer patients. For example, they were willing to wait just under half a week (MRS = 0.45) for every 1% increase in accuracy (i.e. 2.25 weeks for a 5% increase in accuracy).

**Table 4:** Marginal rates of substitution across all attributes

	Lung cancer patients					Colorectal cancer patients			
	Time in a scanner	Time to diagnosis	Radiation dose	Number of additional scans	Accuracy	Time to diagnosis	Radiation dose	Number of additional scans	Accuracy
Numerator of MRS	Willingness to wait in scan (min)	Willingness to wait for diagnosis (weeks)	Willingness to have an additional 1/1000 cancer risk due to radiation exposure	Willingness to have an extra scan	Willingness for a 1% increase in accuracy	Willingness to wait for diagnosis (weeks)	Willingness to have an additional 1/1000 cancer risk due to radiation exposure	Willingness to have an extra scan	Willingness for a 1% increase in accuracy
Time in the scanner	–	− 0.02	− 0.02	− 0.05	0.08	NS	NS	NS	NS
Time to diagnosis	− 45.23	–	− 0.90	− 2.08	3.42	–	− 0.80	− 1.55	2.23
Radiation exposure	− 50.20	− 1.11	–	− 2.31	3.80	− 1.25	–	− 1.94	2.79
Number of additional scans	− 21.77	− 0.48	− 0.43	–	1.65	− 0.64	− 0.51	–	1.44
Accuracy	13.21	0.29	0.26	0.61	–	0.45	0.36	0.70	–

*NS* indicates that the coefficient on time in a scanner is non-significant so the MRS is not computed. The MRS for the willingness for time on the scanner for colorectal cancer patients is not reported because the coefficient on time in a scanner in this group is non-significant. The MRS with regards need for whole body and head to be in a scanner is not reported for either group because in both cases, the coefficient is non-significant

### Predicted probabilities

Figures 3 and 4 detail the predicted probabilities of choosing alternative pathways, compared with a default standard staging pathway for lung (PET-CT plus one additional scan) and colorectal cancer (CT plus one additional scan), respectively. Lung cancer patients were more likely to prefer a WB-MRI-based pathway (probability 0.64) if it was as accurate, required the same total number of scans, and had the same time to diagnosis as the default staging pathway. If the WB-MRI pathway was more accurate, reduced time to diagnosis and/or required fewer scans than the default staging pathway, then the preference for WB-MRI was even stronger. For example, the probability of choosing WB-MRI if it was more accurate than the default pathway was 0.76, rising to 0.89 if WB-MRI was more accurate, reduced time to diagnosis and meant fewer scans. The same patterns were also found for colorectal cancer patients compared with their default staging pathway.

## Discussion

The acceptability or otherwise of WB-MRI as a replacement for current multi-modality pathways is dependent on many factors, most notably diagnostic accuracy and patient acceptability, the latter governed by the contrasting attributes of alternative staging pathways. Using a DCE, we identified those desirable attributes that most influence patient preferences and identified circumstances in which WB-MRI pathways would be preferred by the majority over current staging pathways.

As would be expected, we found that patients generally prefer to wait less time for staging, reduce the cancer risk due to radiation exposure, and undergo fewer scans with greater accuracy. For patients with lung cancer, time to diagnosis was the attribute valued most highly, followed by accuracy, cancer risk from radiation exposure, number of additional scans, and time in a scanner. For patients with colorectal cancer, accuracy was valued most highly, followed by time to diagnosis, cancer risk from radiation exposure, and number of additional scans. Diagnostic accuracy however had a greater influence on the preferences of lung cancer patients who were home-owning or had higher self-rated health. Differences between the two cohorts could therefore reflect demographic and health differences, with colorectal cancer patients reporting lower deprivation, higher educational level, and better health than lung cancer patients. However, the analyses by sub-group within each cohort were exploratory and further research to explore the observed variations would be beneficial.

The length of time in the scanner was a significant factor affecting preferences for patients with lung cancer only, likely because this group finds prolonged scans more challenging. In support, previous data from patients recruited to the Streamline trials have shown that in general, patients with lung cancer find WB-MRI more demanding, often because they cannot hold their breath easily or lie flat for long periods [3].

Cancer risk from radiation exposure significantly influenced the preferences of both cohorts, although was deemed less important than test accuracy and time to diagnosis. The long-term prognosis of the recruited cohort is clearly heavily dependent on their age and underlying primary cancer diagnosis rather than the theoretical small additional cancer risk due to staging investigations. It is likely improved patient education would reduce their perceived importance of ionising radiation exposure, but, nonetheless, long-term survivorship is common for both cancers (particularly colorectal) and exposure to radiation is clearly a legitimate patient concern.

Just over a third of participants were ‘non-traders’, with preferences anchored to a single attribute, most commonly diagnostic accuracy. Traders (who formed the majority) were willing to accept inferior levels of one attribute in turn for improvement in another. For example, the marginal rates of substitution suggest that in return for a 5% improvement in accuracy, patients with colorectal cancer would be prepared to wait an additional 2.25 weeks for their final staging diagnosis and undergo an additional 3.5 scans. Similarly, patients with lung cancer are willing to wait 1.45 weeks for their final staging diagnosis or undergo an additional 3.05 scans for the same 5% accuracy improvement. Many patients were also willing to trade for a reduction in cancer risk due to radiation exposure. For example, to avoid a 1/1000 increase in cancer risk from scan-related radiation exposure, lung cancer patients would wait around 1.11 weeks more for their final diagnosis, despite its likely limited impact on overall prognosis.

This trading of attributes is reflected in overall patient preferences for the various pathway scenarios presented. Patients with lung or colorectal cancer were more likely to prefer a WB-MRI pathway compared with default staging as long as it was as accurate and results in the same scan number and time to diagnosis. As noted above, this suggests that a lack of radiation exposure is believed important by patients. If, however, WB-MRI is more accurate than the standard pathway, reduces time to diagnosis, and/or results in fewer scans, then the preference for WB-MRI is even stronger. Indeed, if WB-MRI is more accurate, reduces time to diagnosis, and results in fewer scans, the probability of preferring it over the standard staging pathway is 0.89 in patients with lung cancer and 0.99 in patients CRC.

Likelihood ratio tests rejected the null hypothesis that none of the attributes were related to preferences. This also provides some reassurance that the problem of multiple comparisons did not arise in our analyses.

Our results were very similar between patients with lung and colorectal cancer, and so we envisage the data could potentially be extrapolated to staging other cancers. However, there were some differences between lung and colorectal cancer patients, which may be in part due to underlying differing comorbidities. It is possible, for example, that patients with pain due to bony metastasis (for example in myeloma) may find prolonged WB-MRI protocols more challenging and this should be investigated. Furthermore, research on patient preferences for WB-MRI vs CT in patients undergoing lymphoma staging showed patients found WB-MRI less unpleasant and less worrisome than CT [20]. The authors attributed their findings to the more invasive preparation required for CT in their scan protocol (patients required intravenous lines and had to consume oral contrast). In our study, WB-MRI protocols required IV gadolinium which may help explain discrepant findings.

The study has limitations. It was powered to detect differences between the two cancer cohorts, but not to detect differences within each cancer type. This may explain non-significant effects across a number of different demographics. The need to enclose the whole body and head did not influence scan preferences when balanced against other test attributes. Previous work has demonstrated that claustrophobia is problematic for many patients undergoing MRI [5, 21]. Patients recruited to the Streamline trials were, by definition, willing to undergo WB-MRI and may therefore not be representative of an unselected cancer patient cohort, particularly given the general prevalence of claustrophobia. Indeed, the majority of participants had already had the WB-MRI scan prior to completing the study. Of note, however, when given a binary choice, lung cancer patients stating an overall preference for standard staging scans preferred less time in a scanner and not to have their whole body and head enclosed compared with those preferring WB-MRI.

Future research could assess what attributes WB-MRI would need to possess in order to appeal to people who are more reluctant to undergo a full body scan.

In conclusion, patients with cancer are willing to trade staging pathway attributes, for example prolonging time to diagnosis, in return for increased accuracy and/or reduced diagnostic radiation exposure. Staging pathways based on first-line WB-MRI are preferred by most patients if they at least match standard pathways for diagnostic accuracy, time to diagnosis, and total scan number. If WB-MRI staging improves any or all these attributes, patient preference is stronger.

## Electronic supplementary material

ESM 1 (DOC 389 kb)

## Abbreviations

CT: Computed tomography
DCE: Discrete choice experiment
MRI: Magnetic resonance imaging
PET-CT: Positron emission tomography
WB-MRI: Whole-body MRI
