# Supplementary material for: Patient preferences for whole-body MRI or conventional staging pathways in lung and colorectal cancer: a discrete choice experiment
Source: Eur Radiol. 2019 Apr 1;29(7):3889–900. doi: 10.1007/s00330-019-06153-4 (PMC6554244; doi:10.1007/s00330-019-06153-4)
Supplement: Supplementary file 1 — (DOC 389 kb) [file 330_2019_6153_MOESM1_ESM.doc]

| Questionnaire A-1 | Time point | Registration |
| --- | --- | --- |

| **We want to know about your preferences for different medical scans** |
| --- |
| You have been invited to take part in a study examining whether whole body magnetic resonance imaging (WB-MRI) is better or quicker than the currently used scans in assessing whether a cancer has spread in people who have just been diagnosed. You have been invited to take part in this study because you are undergoing tests and scans that will provide your doctors with the information they need to correctly diagnose and treat you.  **We understand you may not have been diagnosed with lung cancer, but because your doctor has requested other tests and scans we would still like to invite you to take part.**  We want to know about people’s preferences for different scans so we are inviting you to complete this questionnaire. We are asking approximately 50 patients to complete this questionnaire in 18 hospitals around the UK.  Please be as honest as possible; we really want to know what you think. There are no right or wrong answers. Please answer ALL the questions. Your answers will be treated in strict confidence and are very important for our research.  Please fill in this questionnaire and return it in the enclosed stamped addressed envelope.  If you have any questions or need some help completing the questionnaire please contact:  [information removed]  *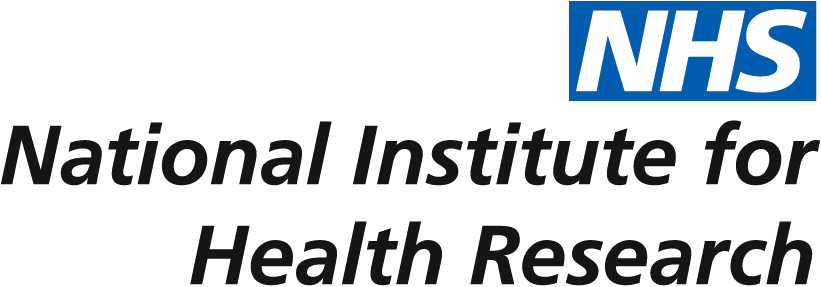*  *This project was funded by the National Institute for Health Research* Health Technology Assessment programme  *(project number 10/68/01)* |

**About the questionnaire:**

The following section contains 9 sets of scan descriptions with 2 different scans in each set. For each set we describe the two scans (A and B) which differ in what you would have to do and/or experience.

We would like you to compare the 2 scans side by side and tell us which one of the two you prefer by selecting A or B each time. This will help us to work out which features of scans are important to you.

After this section, there will be questions about how you are feeling in general and some general background detail on you to help us analyse the results.

**Diagnostic scans for lung cancer can vary in the following ways:**

- **Accuracy:** Scans can vary in how accurate they are. 85% accuracy means for 85 people out of a 100 with lung cancer, the scan will correctly identify both how advanced the cancer is within the lung (local stage), and whether it has or has not spread around the body. The scans described here can correctly identify the local stage and spread of cancer in **85%, 90%** or **95%** of patients.
- **Radiation exposure:** Some scans involve exposure to x-ray radiation, which may increase the lifetime risk of cancer. A one-in-a-thousand increased lifetime risk of cancer means if a thousand people have the scan, one will develop cancer as a result of having had the scan.
- **Being in an enclosed space:** Some scans involve lying in a tunnel where the images are acquired, and means your whole body and head are enclosed.
- **Time to final diagnosis:** This refers to the time from when the first test suggested you may have cancer to the time you get a final full diagnosis including whether the cancer has spread or not, so your doctor can start the correct treatment
- **The number of scans:** This refers to the total number of scans you need from when the first test suggested you may have cancer to the time you get a final full diagnosis including whether the cancer has spread or not, so your doctor can start the correct treatment.

**Example:**

**Read the descriptions of each scan (Scan A and Scan B). If you would rather have Scan A than Scan B you would tick the box under ‘prefer Scan A’ as shown below.**

There are 6 statements listed below. Each statement should be read and then you pick your preferred scan from either Scan A or Scan B.

| **^Ex^ Choice 1** |  | **Scan A** |  | **Scan B** |
| --- | --- | --- | --- | --- |
| You will be in the scanner for: |  | 5 minutes |  | 15 minutes |
| You will know your final full diagnosis after: |  | 2 weeks |  | 4 weeks |
| The lifetime chance of getting cancer because you had a scan that exposed you to radiation is: |  | 3 in 1000 patients |  | 4 in 1000 patients |
| The number of additional scans after this one that you will need before you know your final full diagnosis is: |  | 3 |  | 4 |
| The accuracy of the scans to tell you if the cancer has or has not spread is: |  | Correct in 99 out of 100 patients |  | Correct in 80 out of 100 patients |
| Your whole body including your head will need to be inside the scanner: |  | Yes |  | No |
| Based on the descriptions above, which scan would you prefer? (tick one box only) |  | Prefer Scan A  **☑** |  | Prefer Scan B  **□** |

The person completing this has read the description of Scan A and Scan B and has decided that they prefer the description of Scan A.

**Please look at each set of scan choices (choices 1-9) and choose either Scan A or Scan B for each choice**

| **^A2^ Choice 1** |  | **Scan A** |  | **Scan B** |
| --- | --- | --- | --- | --- |
| You will be in the scanner for: |  | 10 minutes |  | 30 minutes |
| You will know your final full diagnosis after: |  | 5 weeks |  | 1 week |
| The lifetime chance of getting cancer because you had a scan that exposed you to radiation is: |  | 2 in 1000 patients |  | No chance |
| The number of additional scans after this one that you will need before you know your final full diagnosis is: |  | 1 |  | 2 |
| The accuracy of the scans to tell you if the cancer has or has not spread is: |  | Correct in 90 out of 100 patients |  | Correct in 95 out of 100 patients |
| Your whole body including your head will need to be inside the scanner: |  | Yes |  | No |
| Based on the descriptions above, which scan would you prefer? (tick one box only) |  | Prefer Scan A  **□** |  | Prefer Scan B  **□** |

| **^A8^ Choice 2** |  | **Scan A** |  | **Scan B** |
| --- | --- | --- | --- | --- |
| You will be in the scanner for: |  | 30 minutes |  | 60 minutes |
| You will know your final full diagnosis after: |  | 1 week |  | 3 weeks |
| The lifetime chance of getting cancer because you had a scan that exposed you to radiation is: |  | 2 in 1000 patients |  | No chance |
| The number of additional scans after this one that you will need before you know your final full diagnosis is: |  | 2 |  | 0 |
| The accuracy of the scans to tell you if the cancer has or has not spread is: |  | Correct in 90 out of 100 patients |  | Correct in 95 out of 100 patients |
| Your whole body including your head will need to be inside the scanner: |  | No |  | Yes |
| Based on the descriptions above, which scan would you prefer? (tick one box only) |  | Prefer Scan A  **□** |  | Prefer Scan B  **□** |

| **^A7^ Choice 3** |  | **Scan A** |  | **Scan B** |
| --- | --- | --- | --- | --- |
| You will be in the scanner for: |  | 10 minutes |  | 30 minutes |
| You will know your final full diagnosis after: |  | 1 week |  | 3 weeks |
| The lifetime chance of getting cancer because you had a scan that exposed you to radiation is: |  | 1 in 1000 patients |  | 2 in 1000 patients |
| The number of additional scans after this one that you will need before you know your final full diagnosis is: |  | 0 |  | 1 |
| The accuracy of the scans to tell you if the cancer has or has not spread is: |  | Correct in 90 out of 100 patients |  | Correct in 95 out of 100 patients |
| Your whole body including your head will need to be inside the scanner: |  | No |  | Yes |
| Based on the descriptions above, which scan would you prefer? (tick one box only) |  | Prefer Scan A  **□** |  | Prefer Scan B  **□** |

| **^A9^ Choice 4** |  | **Scan A** |  | **Scan B** |
| --- | --- | --- | --- | --- |
| You will be in the scanner for: |  | 10 minutes |  | 30 minutes |
| You will know your final full diagnosis after: |  | 3 weeks |  | 5 weeks |
| The lifetime chance of getting cancer because you had a scan that exposed you to radiation is: |  | 2 in 1000 patients |  | No chance |
| The number of additional scans after this one that you will need before you know your final full diagnosis is: |  | 2 |  | 0 |
| The accuracy of the scans to tell you if the cancer has or has not spread is: |  | Correct in 85 out of 100 patients |  | Correct in 90 out of 100 patients |
| Your whole body including your head will need to be inside the scanner: |  | No |  | Yes |
| Based on the descriptions above, which scan would you prefer? (tick one box only) |  | Prefer Scan A  **□** |  | Prefer Scan B  **□** |

| **^A6^ Choice 5** |  | **Scan A** |  | **Scan B** |
| --- | --- | --- | --- | --- |
| You will be in the scanner for: |  | 10 minutes |  | 30 minutes |
| You will know your final full diagnosis after: |  | 3 weeks |  | 5 weeks |
| The lifetime chance of getting cancer because you had a scan that exposed you to radiation is: |  | No chance |  | 1 in 1000 |
| The number of additional scans after this one that you will need before you know your final full diagnosis is: |  | 2 |  | 0 |
| The accuracy of the scans to tell you if the cancer has or has not spread is: |  | Correct in 95 out of 100 patients |  | Correct in 85 out of 100 patients |
| Your whole body including your head will need to be inside the scanner: |  | Yes |  | No |
| Based on the descriptions above, which scan would you prefer? (tick one box only) |  | Prefer Scan A  **□** |  | Prefer Scan B  **□** |

| **^A5^ Choice 6** |  | **Scan A** |  | **Scan B** |
| --- | --- | --- | --- | --- |
| You will be in the scanner for: |  | 30 minutes |  | 60 minutes |
| You will know your final full diagnosis after: |  | 1 week |  | 3 weeks |
| The lifetime chance of getting cancer because you had a scan that exposed you to radiation is: |  | 1 in 1000 patients |  | 2 in 1000 patients |
| The number of additional scans after this one that you will need before you know your final full diagnosis is: |  | 2 |  | 0 |
| The accuracy of the scans to tell you if the cancer has or has not spread is: |  | Correct in 95 out of 100 patients |  | Correct in 85 out of 100 patients |
| Your whole body including your head will need to be inside the scanner: |  | Yes |  | No |
| Based on the descriptions above, which scan would you prefer? (tick one box only) |  | Prefer Scan A  **□** |  | Prefer Scan B  **□** |

| **^A1^ Choice 7** |  | **Scan A** |  | **Scan B** |
| --- | --- | --- | --- | --- |
| You will be in the scanner for: |  | 30 minutes |  | 60 minutes |
| You will know your final full diagnosis after: |  | 3 weeks |  | 5 weeks |
| The lifetime chance of getting cancer because you had a scan that exposed you to radiation is: |  | No chance |  | 1 in 1000 patients |
| The number of additional scans after this one that you will need before you know your final full diagnosis is: |  | 1 |  | 2 |
| The accuracy of the scans to tell you if the cancer has or has not spread is: |  | Correct in 90 out of 100 patients |  | Correct in 95 out of 100 patients |
| Your whole body including your head will need to be inside the scanner: |  | No |  | Yes |
| Based on the descriptions above, which scan would you prefer? (tick one box only) |  | Prefer Scan A  **□** |  | Prefer Scan B  **□** |

| **^A3^ Choice 8** |  | **Scan A** |  | **Scan B** |
| --- | --- | --- | --- | --- |
| You will be in the scanner for: |  | 60 minutes |  | 10 minutes |
| You will know your final full diagnosis after: |  | 1 week |  | 3 weeks |
| The lifetime chance of getting cancer because you had a scan that exposed you to radiation is: |  | No chance |  | 1 in 1000 patients |
| The number of additional scans after this one that you will need before you know your final full diagnosis is: |  | 1 |  | 2 |
| The accuracy of the scans to tell you if the cancer has or has not spread is: |  | Correct in 95 out of 100 patients |  | Correct in 85 out of 100 patients |
| Your whole body including your head will need to be inside the scanner: |  | No |  | Yes |
| Based on the descriptions above, which scan would you prefer? (tick one box only) |  | Prefer Scan A  **□** |  | Prefer Scan B  **□** |

| **^A4^ Choice 9** |  | **Scan A** |  | **Scan B** |
| --- | --- | --- | --- | --- |
| You will be in the scanner for: |  | 60 minutes |  | 10 minutes |
| You will know your final full diagnosis after: |  | 1 week |  | 3 weeks |
| The lifetime chance of getting cancer because you had a scan that exposed you to radiation is: |  | 2 in 1000 patients |  | No chance |
| The number of additional scans after this one that you will need before you know your final full diagnosis is: |  | 1 |  | 2 |
| The accuracy of the scans to tell you if the cancer has or has not spread is: |  | Correct in 85 out of 100 patients |  | Correct in 90 out of 100 patients |
| Your whole body including your head will need to be inside the scanner: |  | Yes |  | No |
| Based on the descriptions above, which scan would you prefer? (tick one box only) |  | Prefer Scan A  **□** |  | Prefer Scan B  **□** |

| **Have you had any of the following tests yet?** | | | |
| --- | --- | --- | --- |
|  | Yes | No | Not sure |
| Whole body MRI (this scan takes about an hour and looks at your whole body) | **□** | **□** | **□** |
| PET-CT (for this scan you are injected with a small amount of radioactive tracer and the scan is performed about an hour later) | **□** | **□** | **□** |
| CT scan (this scan takes about 10 minutes and you are injected with some X-Ray dye into a vein during it) | **□** | **□** | **□** |
| Any other scan or test, please specify ……………………………………………………………………………………………………………………………………………………………………………………………………………………………………………………………………………………………………………………………………………………………………………... | | | |

| **If you had to have JUST ONE of the tests which one would you prefer? Please circle or underline the one you would choose.** | | |
| --- | --- | --- |
| Whole body MRI | **OR** | PET-CT |

| **How are you feeling right now?** | | | | | |
| --- | --- | --- | --- | --- | --- |
| Below is a list of words that describe different feelings you might be experiencing right now. We are interested in your GENERAL mood at the moment. Please read EACH word and tick (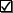) the box that best indicates the extent to which you feel this way **right now**. Have a look at the EXAMPLE below: | | | | | |
| **EXAMPLE:** Consider the word ‘sleepy’ | | | | | |
| If you are NOT feeling sleepy right now you should tick (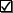) ‘very slightly or not at all’. | | | | | |
|  | Very slightly or not at all | A little | Moderately | Quite a bit | Extremely |
| Sleepy | 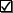 | **□** | **□** | **□** | **□** |
| Or if you feel MODERATELY sleepy you should tick (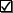) ‘moderately’. | | | | | |
|  | Very slightly or not at all | A little | Moderately | Quite a bit | Extremely |
| Sleepy | **□** | **□** |  | **□** | **□** |
| Please read **EACH** word carefully. If a word does not apply or make sense to you, please tick ‘very slightly or not at all’. It is important that you tick a box (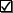) for **EVERY** word. | | | | | |
|  | Very slightly or not at all | A little | Moderately | Quite a bit | Extremely |
| Active | **□** | **□** | **□** | **□** | **□** |
| Distressed | **□** | **□** | **□** | **□** | **□** |
| Excited | **□** | **□** | **□** | **□** | **□** |
| Upset | **□** | **□** | **□** | **□** | **□** |
| Strong | **□** | **□** | **□** | **□** | **□** |
| Guilty | **□** | **□** | **□** | **□** | **□** |
| Scared | **□** | **□** | **□** | **□** | **□** |
| Hostile | **□** | **□** | **□** | **□** | **□** |
| Enthusiastic | **□** | **□** | **□** | **□** | **□** |
| Proud | **□** | **□** | **□** | **□** | **□** |
| Irritable | **□** | **□** | **□** | **□** | **□** |
| Alert | **□** | **□** | **□** | **□** | **□** |
| Ashamed | **□** | **□** | **□** | **□** | **□** |
| Inspired | **□** | **□** | **□** | **□** | **□** |
| Nervous | **□** | **□** | **□** | **□** | **□** |
| Determined | **□** | **□** | **□** | **□** | **□** |
| Attentive | **□** | **□** | **□** | **□** | **□** |
| Jittery (on edge) | **□** | **□** | **□** | **□** | **□** |
| Interested | **□** | **□** | **□** | **□** | **□** |
| Afraid | **□** | **□** | **□** | **□** | **□** |

**These are some general questions about your outlook on life and general health because these can affect people’s attitude towards medical scans.**

| **How is your health in general?** | | | | |
| --- | --- | --- | --- | --- |
| Very bad | Bad | Fair | Good | Very good |
| **□** | **□** | **□** | **□** | **□** |

| **Do you have any of the following problems at the moment? Please tick any that apply.** | | |
| --- | --- | --- |
| Unexplained weight loss | Persistent chest infection | Cough that does not go away |
| **□** | **□** | **□** |
| Shortness of breath when you are sitting up | Shortness of breath when you are lying down | Persistent chest pain |
| **□** | **□** | **□** |
| Persistent shoulder pain | Coughing up blood | Ache or pain when breathing |
| **□** | **□** | **□** |
| Loss of appetite | Painful cough | Persistent tiredness |
| **□** | **□** | **□** |

| **Your thoughts about the future** | | | | | |
| --- | --- | --- | --- | --- | --- |
|  | Strongly disagree | Disagree | Not sure | Agree | Strongly agree |
| I think about the future and this influences my behaviour today | **□** | **□** | **□** | **□** | **□** |
| I leave the future to take care of itself | **□** | **□** | **□** | **□** | **□** |
| I’m prepared to make sacrifices now for benefit in the long run | **□** | **□** | **□** | **□** | **□** |
| I prefer to think of the ‘here and now’ rather than the future | **□** | **□** | **□** | **□** | **□** |

| **You will find below a series of statements which describe how people may react to the uncertainties of life. Please use the scale below to describe to what extent each item is characteristic of you. Please circle a number (1 to 5) that describes you best.** | | | | | |
| --- | --- | --- | --- | --- | --- |
|  | Not at all characteristic of me |  | Somewhat characteristic of me |  | Entirely characteristic of me |
| My mind can’t be relaxed if I don’t know what will happen tomorrow | **1** | **2** | **3** | **4** | **5** |
| Uncertainty makes life intolerable | **1** | **2** | **3** | **4** | **5** |
| Uncertainty makes me uneasy, anxious or stressed | **1** | **2** | **3** | **4** | **5** |
| Uncertainty keeps me from sleeping soundly | **1** | **2** | **3** | **4** | **5** |

**Finally, here are a few questions about you to help us analyse the results of the survey.**

| **Please write your age** | | | | | | | | | | | |
| --- | --- | --- | --- | --- | --- | --- | --- | --- | --- | --- | --- |
| **Are you…** | | |  | |  | | | **□** Male | | | **□** Female |
| **Are you currently:** | | | | | | | | | | | |
| **□** | Employed full-time | | | | | **□** | | Retired | | | |
| **□** | Employed part-time | | | | | **□** | | Student | | | |
| **□** | Unemployed | | | | | **□** | | Disabled or too ill to work | | | |
| **□** | Full-time homemaker | | | | | **□** | | Self-employed | | | |
|  |  | | | | | **□** | | Do not wish to answer | | | |
| **What is your marital status?** | | | | | | | | | | | |
| Single | | Married | | Cohabiting / living with partner | | | Divorced / separated | | Widowed | Do not wish to answer | |
| **□** | | **□** | | **□** | | | **□** | | **□** | **□** | |
| **Does your household own or rent the accommodation you live in? Tick ONE box only** | | | | | | | | | | | |
| Owns  outright | | Owns with a mortgage or loan | | Part owns and part rents | | | Rents (with or without housing benefit) | | Lives here rent free | Do not wish to answer | |
| **□** | | **□** | | **□** | | | **□** | | **□** | **□** | |

| **In total, how many cars or vans are owned, or are available for use, by members of your household. Include any company car(s) or van(s) available for private use.** | | | | |
| --- | --- | --- | --- | --- |
| None | 1 | 2 | 3 or more | Do not wish to answer |
| **□** | **□** | **□** | **□** | **□** |

| **Which of these qualifications do you have? Tick any that apply.** | | | |
| --- | --- | --- | --- |
| **□** | 1 – 4 O levels / CSEs / GCSEs (any grades), Entry Level, Foundation Diploma | **□** | NVQ Level 1, Foundation GNVQ, Basic Skills |
| **□** | 5+ O levels (passes) / CSEs (grade 1) / GCSEs (grades A*- C), School Certificate, 1 A level / 2 – 3 AS levels / VCEs, Higher Diploma | **□** | NVQ Level 2, Intermediate GNVQ, City and Guilds Craft, BTEC First / General Diploma, RSA Diploma |
| **□** | Apprenticeship | **□** | 2+ A levels / VCEs, 4+ AS levels, Higher School Certificate, Progression / Advanced Diploma |
| **□** | NVQ Level 3, Advanced GNVQ, City and Guilds Advanced Craft, ONC, OND, BTEC National, RSA Advanced Diploma | **□** | Degree (for example BA, BSc), Higher degree (for example MA, PhD, PGCE) |
| **□** | NVQ Level 4 – 5, HNC, HND, RSA Higher Diploma, BTEC Higher Level | **□** | Professional qualifications (for example teaching, nursing, accountancy) |
| **□** | Other vocational / work-related qualifications | **□** | Foreign qualifications |
| **□** | No qualifications | **□** | Do not wish to answer |

| Do you have any of the following diseases? | | | | | | | |
| --- | --- | --- | --- | --- | --- | --- | --- |
| Heart or Vascular Disease | | Diabetes | | Epilepsy | | Stroke | Arthritis |
| □ | | □ | | □ | | □ | □ |
| Asthma | Mental or Emotional Disorder | | Any other illness (excluding cancer)? | |  | | |
| □ | □ | | □ | | Please specify___________________ | | |

| **What is your ethnic group?** | | | | | | | | | | |
| --- | --- | --- | --- | --- | --- | --- | --- | --- | --- | --- |
| **A. White** | | | | | | | | | | |
| English / Welsh / Scottish / Northern Irish / British | | Irish | | | Gypsy or Irish Traveller | | | | Any other White background | |
| **□** | | **□** | | | **□** | | | | **□** | |
| **B. Mixed / multiple ethnic groups** | | | | | | | | | | |
| White and Black Caribbean | | White and Black African | | | White and Asian | | | | Any other Mixed / multiple ethnic background | |
| **□** | | **□** | | | **□** | | | | **□** | |
| **C. Asian / Asian British** | | | | | | | | | | |
| Indian | Pakistani | | | Bangladeshi | | | Chinese | | | Any other Asian background |
| **□** | **□** | | | **□** | | | **□** | | | **□** |
| **D. Black / African / Caribbean / Black British** | | | | | | | | | | |
| African | | | Caribbean | | | | | Any other Black / African / Caribbean background | | |
| **□** | | | **□** | | | | | **□** | | |
| **E. Other ethnic group** | | | | | |  | | | | |
| Arab | | | | | | Any other ethnic group | | | | |
| **□** | | | | | | **□** | | | | |
| **F. Do not wish to answer** | | | | | |  | | | | |
| Do not wish to answer | | | | | |  | | | | |
| **□** | | | | | |  | | | | |

| **If you have any further comments regarding your experiences or comments about this questionnaire, please write them here.** |
| --- |

**THANK YOU VERY MUCH FOR YOUR HELP WITH THIS RESEARCH**

**Supplementary data**

**Sample size calculation**

Sample size requirements for DCEs are not straightforward and are frequently unreported (1). Our calculations were based on the formula (500*c)/(t*a) where t = the number of sets of choices, a = the number of scenarios to choose between in each choice, and c = the largest number of levels for any one attribute (2). Thus our target sample size was 42 patients for each cancer type.

**Questionnaire A for lung cancer patients**

Appended separately.

**Statistical analysis in full**

Descriptive statistics for participant characteristics were compared between colorectal and lung cancer cohorts using χ^2^ tests for categorical variables and independent t-tests for continuous variables.

DCE data were analysed using a conditional logit regression model (fixed effects logit) where the outcome was test preference (scan A or B) and the variables in the equation were the individual attributes. We ran the model on the whole sample, as well as stratifying by the two trials, and by patient characteristics.

We used the regression coefficients to compute marginal rates of substitution (MRS).

We also used the regression analysis results to calculate the predicted probabilities of choosing alternative pathways (for example based on WB-MRI), compared to a default standard staging pathway. The selected default standard pathway was PET-CT plus one additional scan (lung cancer), or CT plus 1 additional scan (colorectal cancer). Attributes we defined as describing the default standard staging pathways in lung and colorectal cancer are listed in the legends of figures 3 and 4. We compared default staging pathways to alternative pathways with varying attribute levels based around PET-CT, CT, and WB-MRI. We considered several scenarios for WB-MRI based pathways, although fixed the following attributes; i). 60 minutes in the scanner, ii) no risk of cancer from radiation exposure, and iii) requirement for the whole body and head to be enclosed. We then varied combinations of time to diagnosis, number of additional scans and accuracy of WB-MRI individually and jointly. Non-traders were included in the analysis.

All data were analysed using SPSS version 24 and Stata version 13

Within each cohort, we also ran regression models stratifying by gender, age (≥66 years (median age)/<66 years), presence of comorbidities (yes/no), employment status (employed, self-employed or full-time homemaker / retired / unemployed, disabled or too ill to work), home ownership (yes/no), education (no qualifications/qualifications below degree level/degree level or equivalent), marital status (married or cohabiting / single / divorced, separated or widowed), self-rated health (very poor, poor or fair / good or very good), positive mood (≥27 PANAS (median score)/<27), and prior test preference (WB-MRI/standard tests). We tested for differences in preferences between sub-groups using χ^2^ tests. Within each cohort, there was no significant influence on preferences according to gender, age, comorbidities, employment status, marital status and positive mood. For patients with colorectal cancer there were no significant preference differences when stratifying by home ownership, education and self-rated health. Significant effects for lung are tabulated below.

**Table A1: Results of conditional logit regression analysis: lung cancer patients stratified by home ownership (yes/no)**

|  |  | **All lung cancer patients** | **Lung cancer patients; home ownership= yes** | **Lung cancer patients; home ownership= no** |  |
| --- | --- | --- | --- | --- | --- |
| **Attributes** | **Levels** | **Coefficient (95% CI)** | **Coefficient (95% CI)** | **Coefficient (95% CI)** | **P-value**^b^ |
| Time in scanner | Minutes | -0.008 (-0.014, -0.002) | -0.010 (-0.020, 0.0003) | -0.011 (-0.023, 0.002)^a^ | 0.89 |
| Time to diagnosis | Weeks | -0.372 (-0.449, -0.295) | -0.469 (-0.603, -0.334) | -0.391 (-0.539, -0.244) | 0.45 |
| Radiation dose | Risk of cancer (/1000) | -0.413 (-0.551, -0.274) | -0.582 (-0.809, -0.354) | -0.391 (-0.626, -0.156) | 0.25 |
| Number of additional scans | Number | -0.179 (-0.330, -0.028) | -0.063 (-0.191, 0.318)^a^ | -0.354 (-0.654, -0.054) | 0.03 |
| Accuracy | Percentage | 0.109 (0.079, 0.138) | 0.181 (0.129, 0.233) | 0.077 (0.035, 0.119) | 0.01 |
| Need for whole body and | No | - | - | - |  |
| head to be in scanner | Yes | 0.017 (-0.190, 0.224)^a^ | 0.026 (-0.316, 0.367)^a^ | 0.146 (-0.201, 0.493)^a^ | 0.63 |
| Observations/respondents |  | 1230/72 | 582/34 | 576/33 | 0.02 |

NB: Different attributes do not have the same unit of change so cannot be directly compared with one another

CI = confidence interval

^a^ Coefficient not significantly different from zero; all other coefficients significant at P-value<0.05.

^b^ P-values are from χ^2^ tests that coefficients are equal for the two sub-groups. P-values<0.05 indicate coefficients are significantly different between groups. P-value in bottom row is for joint test across all coefficients.

**Table A2: Results of conditional logit regression analysis: lung cancer patients stratified by education (no qualifications or qualifications below degree level / degree level or equivalent)**

|  |  | **All lung cancer patients** | **Lung cancer patients; education= degree level or equivalent** | **Lung cancer patients; education= no qualifications or qualifications below degree level** |  |
| --- | --- | --- | --- | --- | --- |
| **Attributes** | **Levels** | **Coefficient (95% CI)** | **Coefficient (95% CI)** | **Coefficient (95% CI)** | **P-value**^b^ |
| Time in scanner | Minutes | -0.008 (-0.014, -0.002) | -0.002 (-0.014, 0.011)^a^ | -0.011 (-0.020, -0.003) | 0.24 |
| Time to diagnosis | Weeks | -0.372 (-0.449, -0.295) | -0.369 (-0.541, -0.198) | -0.432 (-0.540, -0.324) | 0.56 |
| Radiation dose | Risk of cancer (/1000) | -0.413 (-0.551, -0.274) | -0.522 (-0.834, -0.210) | -0.356 (-0.532, -0.180) | 0.36 |
| Number of additional scans | Number | -0.179 (-0.330, -0.028) | -0.041 (-0.282, 0.364)^a^ | -0.271 (-0.479, -0.064) | 0.11 |
| Accuracy | Percentage | 0.109 (0.079, 0.138) | 0.190 (0.115, 0.264) | 0.092 (0.054, 0.130) | 0.01 |
| Need for whole body and | No | - | - | - |  |
| head to be in scanner | Yes | 0.017 (-0.190, 0.224)^a^ | -0.252 (-0.695, 0.190)^a^ | 0.208 (-0.070, 0.486)^a^ | 0.09 |
| Observations/respondents |  | 1230/72 | 310/19 | 776/44 | 0.04 |

NB: Different attributes do not have the same unit of change so cannot be directly compared with one another

CI = confidence interval

^a^ Coefficient not significantly different from zero; all other coefficients significant at P-value<0.05.

^b^ P-values are from χ^2^ tests that coefficients are equal for the two sub-groups. P-values<0.05 indicate coefficients are significantly different between groups. P-value in bottom row is for joint test across all coefficients.

**Table A3: Results of conditional logit regression analysis: lung cancer patients stratified by self-rated health (very poor, poor or fair / good or very good)**

|  |  | **All lung cancer patients** | **Lung cancer patients; self-rated health = good or very good** | **Lung cancer patients; self-rated health = very poor, poor or fair** |  |
| --- | --- | --- | --- | --- | --- |
| **Attributes** | **Levels** | **Coefficient (95% CI)** | **Coefficient (95% CI)** | **Coefficient (95% CI)** | **P-value**^b^ |
| Time in scanner | Minutes | -0.008 (-0.014, -0.002) | -0.010 (-0.022, 0.002)^a^ | -0.009 (-0.016, -0.001) | 0.81 |
| Time to diagnosis | Weeks | -0.372 (-0.449, -0.295) | -0.455 (-0.607, -0.304) | -0.342 (-0.445, -0.238) | 0.22 |
| Radiation dose | Risk of cancer (/1000) | -0.413 (-0.551, -0.274) | -0.119 (-0.348, 0.109)^a^ | -0.613 (-0.810, -0.415) | 0.01 |
| Number of additional scans | Number | -0.179 (-0.330, -0.028) | -0.332 (-0.619, -0.044) | -0.095 (-0.291, 0.100)^a^ | 0.18 |
| Accuracy | Percentage | 0.109 (0.079, 0.138) | 0.151 (0.103, 0.200) | 0.080 (0.043, 0.116) | 0.02 |
| Need for whole body and | No | - | - | - |  |
| head to be in scanner | Yes | 0.017 (-0.190, 0.224)^a^ | 0.184 (-0.204, 0.572)^a^ | -0.055 (-0.323, 0.212)^a^ | 0.32 |
| Observations/respondents |  | 1230/72 | 534/29 | 678/37 | 0.01 |

NB: Different attributes do not have the same unit of change so cannot be directly compared with one another

CI = confidence interval

^a^ Coefficient not significantly different from zero; all other coefficients significant at P-value<0.05.

^b^ P-values are from χ^2^ tests that coefficients are equal for the two sub-groups. P-values<0.05 indicate coefficients are significantly different between groups. P-value in bottom row is for joint test across all coefficients.

**Table A4: Results of conditional logit regression analysis: lung cancer patients stratified by test preference (WB-MRI / standard tests)**

|  |  | **All lung cancer patients** | **Lung cancer patients; prior test preference = WB-MRI** | **Lung cancer patients; prior test preference = standard tests** |  |
| --- | --- | --- | --- | --- | --- |
| **Attributes** | **Levels** | **Coefficient (95% CI)** | **Coefficient (95% CI)** | **Coefficient (95% CI)** | **P-value**^b^ |
| Time in scanner | Minutes | -0.008 (-0.014, -0.002) | 0.008 (-0.008, 0.024)^a^ | -0.014 (-0.025, -0.003) | 0.03 |
| Time to diagnosis | Weeks | -0.372 (-0.449, -0.295) | -0.340 (-0.523, -0.157) | -0.262 (-0.409, -0.115) | 0.51 |
| Radiation dose | Risk of cancer (/1000) | -0.413 (-0.551, -0.274) | -0.294 (-0.809, 0.221)^a^ | -0.251 (-0.692, 0.190)^a^ | 0.90 |
| Number of additional scans | Number | -0.179 (-0.330, -0.028) | -0.512 (-1.029, 0.004)^a^ | -0.386 (-0.813, 0.040)^a^ | 0.71 |
| Accuracy | Percentage | 0.109 (0.079, 0.138) | 0.092 (-0.020, 0.204)^a^ | -0.008 (-0.116, 0.100)^a^ | 0.21 |
| Need for whole body and | No | - | - | - |  |
| head to be in scanner | Yes | 0.017 (-0.190, 0.224)^a^ | 0.359 (-0.152, 0.870)^a^ | -0.722 (-1.269, -0.176) | 0.01 |
| Observations/respondents |  | 1230/72 | 234/15 | 292/18 | 0.01 |

NB: Different attributes do not have the same unit of change so cannot be directly compared with one another

CI = confidence interval

^a^ Coefficient not significantly different from zero; all other coefficients significant at P-value<0.05.

^b^ P-values are from χ^2^ tests that coefficients are equal for the two sub-groups. P-values<0.05 indicate coefficients are significantly different between groups. P-value in bottom row is for joint test across all coefficients.

**Table A5: Participants unwilling to trade attributes off against one another (Numbers are N (percent))**

| **Attribute** | **All patients** | **Lung cancer patients** | **Colorectal cancer patients** |
| --- | --- | --- | --- |
| Accuracy: non traders are those who always prefer more accurate tests | 18 (13) | 7 (10) | 11 (17) |
| Time to diagnosis: non traders are those who always prefer shorter time to diagnosis | 16 (12) | 7 (10) | 9 (14) |
| Radiation: non traders are those who always prefer less radiation | 11 (8) | 7 (10) | 4 (6) |
| Scan time: non traders are those who always prefer shorter scan time | 2 (1) | 1 (1) | 1 (2) |
| Scan number: non traders are those who always prefer fewer scans | 2 (1) | 0 (0) | 2 (3) |
| Enclosed: non traders are those who always prefer non enclosure of head and body | 2 (1) | 1 (1) | 1 (2) |
| All | 51 (37) | 23 (32) | 28 (42) |
